# An expression based REST signature predicts patient survival and therapeutic response for glioblastoma multiforme

**DOI:** 10.1038/srep34556

**Published:** 2016-10-04

**Authors:** Jianfeng Liang, Qinghua Meng, Wanni Zhao, Pan Tong, Ping Li, Yuanli Zhao, Xiaodong Zhao, Hua Li

**Affiliations:** 1 Department of Neurosurgery, Peking University International Hospital, Beijing, 102206, China; 2 Department of Nutrition, Jinan Central Hospital Affiliated to Shandong University, Jinan, Shandong Province, 250013, China; 3 Peking University China-Japan Friendship School of Clinical Medicine, Beijing, 100029, China; 4 Department of Bioinformatics and Computational Biology, The University of Texas M. D. Anderson Cancer Center, Houston, TX 77030, USA; 5 Department of Hematology, Tongji Hospital of Tongji University, Shanghai, 200065, China; 6 Department of Neurosurgery, Beijing Tiantan Hospital, Capital Medical University, Beijing, 100050, China; 7 Bio-ID Center, School of Biomedical Engineering, Shanghai Jiao Tong University, Shanghai, 200240, China

## Abstract

Proper regulation of neuronal gene expression is crucial for the development and differentiation of the central nervous system. The transcriptional repressor REST (repressor element-1 silencing transcription factor) is a key regulator in differentiation of pluripotent stem cells to neuronal progenitors and mature neurons. Dysregulated REST activity has been implicated in various diseases, among which the most deadly is glioblastoma multiforme (GBM). Here we have developed an expression-based REST signature (EXPREST), a device providing quantitative measurements of REST activity for GBM tumors. EXPREST robustly quantifies REST activity (REST score) using gene expression profiles in absence of clinic-pathologic assessments of REST. Molecular characterization of REST activity identified global alterations at the DNA, RNA, protein and microRNA levels, suggesting a widespread role of REST in GBM tumorigenesis. Although originally aimed to capture REST activity, REST score was found to be a prognostic factor for overall survival. Further, cell lines with enhanced REST activity was found to be more sensitive to IGF1R, VEGFR and ABL inhibitors. In contrast, cell lines with low REST score were more sensitive to cytotoxic drugs including Mitomycin, Camptothecin and Cisplatin. Together, our work suggests that therapeutic targeting of REST provides a promising opportunity for GBM treatment.

Glioblastoma multiforme (GBM) is the most common and malignant primary brain tumor in adults^1^. Upon initial diagnosis of GBM, standard-of-care treatment is largely palliative and encompasses surgical resection, radiotherapy and adjuvant chemotherapy with temozoomide^2^. Following standard treatments, recurrence usually occurs with a median time to recurrence of 7 months^3^. Over the last decade, a variety of treatments have been explored but the success is rather limited. Most patients diagnosed with GBM die within a year and less than 10% of patients are able to survive after 5 years^4^. This is in part due to tumor heterogeneity, location in hard to reach region, and rapid, aggressive tumor relapse. There is a pressing need to better understand brain tumor biology and develop more effective therapies.

The RE1-silencing transcription factor REST, also known as neuron-restrictive silencer factor NRSF, has been reported as a master regulator of neurogenesis and neuronal differentiation^5^. REST binds to a highly conserved 23 bp motif known as RE1 (repressor element 1) located in a large number of genes encoding neuronal traits and silences their transcription by recruiting several cellular cofactors including Co-REST, N-CoR, and mSin3A forming a REST repressor complex^6^. REST has been shown to coordinate neural induction and differentiation processes both *in vivo* and *in vitro* during neurogenesis^7^. Dysregulated REST function has been implicated in several cancers including breast cancer^8^, colorectal cancer^9^, small cell lung cancer^10^, neuroblastoma^11^, and medulloblastoma^12^. Recently, REST has been reported to control self-renewal and tumorigenic competence of glioblastoma (GBM) cell lines^13^.

Based on a published REST signature consisting of 24 downstream targets of REST^14^, we previously discovered that REST activity predicted drug sensitivity in neuroblastoma cell lines and was a prognostic biomarker for neuroblastoma tumor stage^15^. However, the functional impact of REST remains uncharacterized for gliomas, especially GBM. Using multidimensional genomic, preclinical and clinical data, we developed an expression based REST signature and assessed its association with transcriptomics, genomics, drug sensitivity, and clinical outcome for GBM in this article.

## Results

### Development of the EXPREST signature

We started with a previously published REST signature^14^ which was developed using gene expression array data from human embryonic kidney-293, mammary epithelial MCF10, and breast cancer T-47D cell lines. This signature was later found to apply well in neuroblastoma and predicted drug sensitivity and tumor stage^15^. However, directly applying the published signature to GBM data sets showed contradictory results and invalidated its utility for GBM. In particular, the published signature genes supposedly to be transcriptionally repressed by REST and thus should have anti-correlation with REST mRNA level. In contrast to this hypothesis, the signature genes did not correlate well with REST mRNA level and manifested opposite correlation directions in GBM cell line data (Supplementary Table S1). As a result, the derived REST score cannot capture REST activity in GBM samples. This is likely due to the fundamental differences of tumor biology between GBM and other cancers. Since there is no GBM specific REST signature published to date, it is therefore important to derive an appropriate REST signature for GBM tumors.

**Figure 1:**
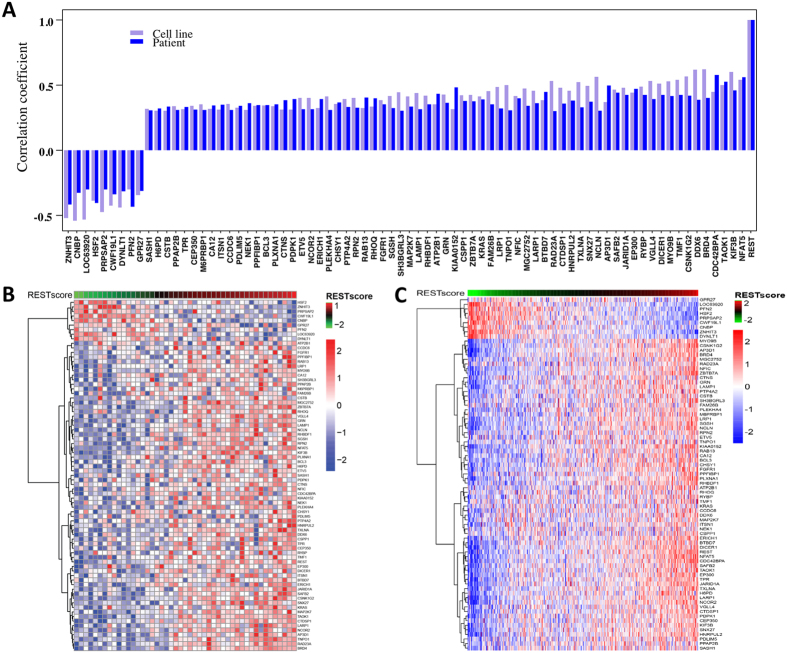
The EXPREST signature accurately quantifies REST potential in both cell lines and patient tumors. (**A**) Pearson’s correlation between EXPREST signature genes and REST expression. Consistent correlations were observed in both cell lines and patient tumors. (**B**) The expression pattern of signature genes in CNS cell lines. Cell lines in the columns were ordered by REST score in increasing order. Genes were clustered with Ward’s linkage rule using 1-Pearson correlation as distance metric. (**C**) The expression pattern of signature genes in TCGA GBM patients.

By leveraging both the cell line and patient expression profiles, we derived an expression-based REST signature (**EXPREST**) using a seed guided approach (see Methods for details). The signature contained 68 genes with positive correlations and 9 genes with negative correlations to REST expression. Genes in our REST signature behaved consistently between cell line and patient data, demonstrating its general applicability to GBM tumors (Fig. 1A). There was a clear pattern of REST signature expression demarked by two clusters of genes in both cell lines (Fig. 1B) and GBM patients (Fig. 1C). Despite of the variations of expression observed in individual signature genes, the REST score computed from the EXPREST signature (See methods) was able to characterize the overall pattern of REST activity. The new signature readily identified a subset of tumors with low REST activity (low REST score) and another set of tumors with high REST activity (high REST score).

### Functional annotation of the REST signature

The EXPREST signature consisted of genes involved in different functional modules. A summary of enriched pathways identified by Ingenuity Pathway Analysis is provided in Supplementary Table S2. The most significantly enriched pathway was neurotrophin/TRK signaling (Hypergeometric test, adjusted p = 0.0048). Neurotrophins initially identified in sensory and sympathetic neurons have been shown to control many aspects of neuronal survival, development and function in the peripheral and central nervous systems^16^. Neurotrophins and their receptors frequently expressed in malignant gliomas and was found to contribute to glioma invasion and proliferation^17^.

It was interesting to note that genes involved in Huntington’s disease were enriched in this signature, namely MAP2K7, PDPK1, NCOR2, EP300, and REST. Huntington’s disease is a neurodegenerative genetic disorder. Previous findings show that REST as a transcriptional repressor inhibits neuronal differentiation in non-neural cells^18^ and maintains stemness of neural stem cells^19^. The enrichment of genes involved in Huntington’s disease shed some light on the multifaceted nature of REST activity in human diseases.

### REST activity is linked to tumorigenesis and mesenchymal properties

**Figure 2:**
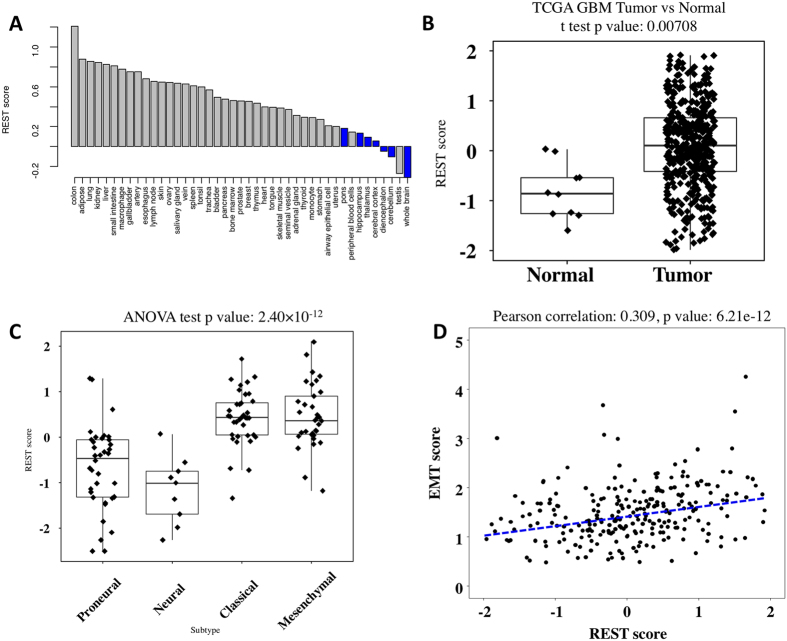
REST activity is linked to tumorigenesis and mesenchymal properties. (**A**) Distribution of REST scores in healthy tissues. Blue bars indicated tissues dissected from different regions of the brain. Brain tissues had much lower REST activity compared to other healthy tissues (t test p = 1.11 × 10^−5^). (**B**) REST score in TCGA GBM tumors and adjacent normal tissues. GBM tumors had elevated REST activity compared to normal tissue (t test p = 0.0071). (**C**) Increased REST activity was associated with the Mesenchymal and Classical subtypes (ANOVA test *P* = 2.40 × 10^−12^). (**D**) REST activity was associated with EMT score (Pearson r = 0.309, *P* = 6.21 × 10^−12^).

We further investigated the spectrum of REST activity across healthy human tissues. Among the 43 healthy tissue samples, brain samples dissected from different brain regions (N = 7) had significantly lower REST activity (t test p = 1.11 × 10^−5^, effect size  = 2.06), which was in concordance with previous findings that REST activity is down-regulated in brain when embryonic stem cells develop into neural progenitors^20^ (Fig. 2A). Since REST activity is repressed in normal brain tissue, we hypothesized that dysregulation of REST might contribute to the tumorigenesis of GBM. We therefore compared REST score of GBM tumors to adjacent normal tissue using TCGA data. Based on 473 tumors and 10 adjacent normal tissue samples, we found that REST activity was significantly upregulated in GBM (t test p = 0.0071) compared to normal tissue, suggesting elevated REST activity might be an important event during tumorigenesis of GBM (Fig. 2B).

It has been found that GBM tumors are quite heterogeneous at the molecular level. Recently, an integrated analysis with multidimensional genomic data identified four molecular subtypes, namely, Proneural, Neural, Classical, and Mesenchymal among GBM patients^21^. To investigate the molecular underpinnings of our new signature, we compared the REST activity among the four molecular subtypes. Fig. 2C showed that different molecular subtypes had significantly different REST activity (ANOVA test p = 2.40 × 10^−12^). There was no difference in REST activity between Proneural and Neural subtype (p = 0.283) or between Classical and Mesenchymal subtypes (p = 0.996) using Tukey’s Highest Significance Test. However, Proneural and Neural subtypes had significantly lower REST activity compared to the Classical and Mesenchymal subtypes. Previous studies showed that epithelial to mesenchymal transition (EMT) played important roles in tumor progression, metastasis, and therapeutic resistance^22^,^23^,^24^. Since increased REST activity was observed in the Mesenchymal subtype, we thus sought to formally evaluate if REST activity was associated with EMT using a recently published EMT signature^25^. Fig. 2D showed that high REST activity was strongly associated with high EMT score (Pearson r = 0.309, p = 6.21 × 10^−12^), a metric measuring the potential of EMT^25^. The enrichment of Mesenchymal subtype and strong association to EMT demonstrated that dysregulated REST might be a co-occurring event in dedifferentiated and transdifferentiated GBM tumors.

### Global transcriptomic changes associated with REST activity

**Figure 3:**
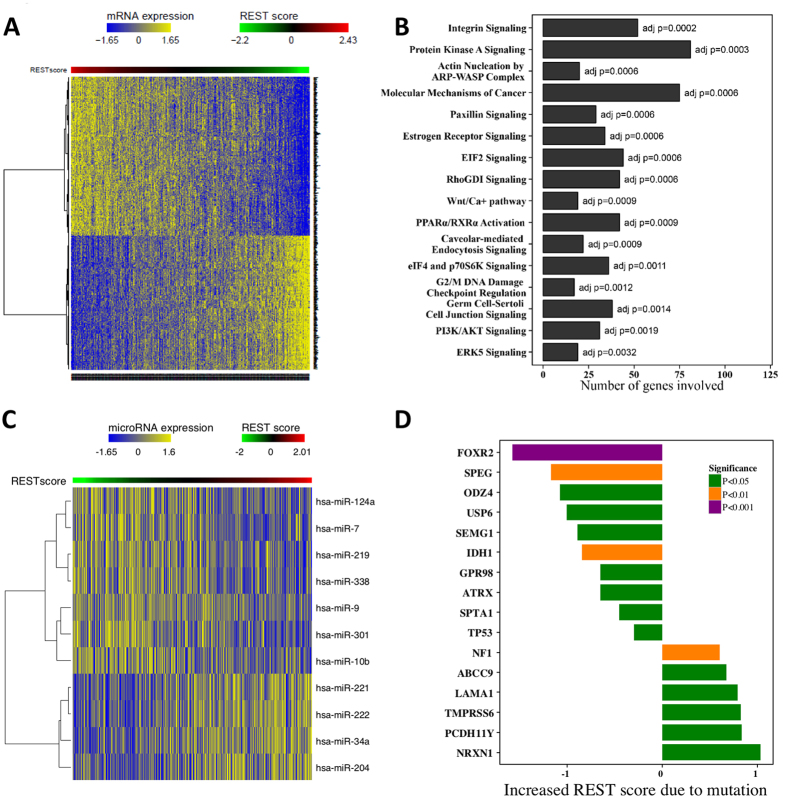
Genomic characterization of the EXPREST signature. (**A**) Hierarchical clustering of TCGA GBM expression data using genes associated with REST score. At FDR = 0.01, 9533 genes were identified. (**B**) Enriched pathways in genes significantly associated with REST score. Adjusted P values were calculated using the Benjamini-Hochberg procedure. (**C**) miRNAs associated with REST activity. (**D**) Mutations associated with REST activity. X-axis showed difference of mean REST score between mutated samples and wildtype samples.

Using the EXPREST signature, we evaluated the impact of increased REST activity on global gene expression pattern, miRNA difference and mutational landscape. We assessed the impact of increased REST activity on transcriptome by correlating REST score with mRNA expression data. A total of 9533 genes were significantly associated with REST score at FDR = 0.01, demonstrating the widespread expression change exerted by REST regulation. Genes associated with REST preserved consistent expression pattern (Fig. 3A). The widespread mRNA expression regulation was found to be enriched within several pathways including integrin signaling, EIF2 signaling, protein kinase A (PKA) signaling, PI3K/Akt signaling, G2/M DNA damage checkpoint regulation and Wnt/Ca+ pathway (Fig. 3B). Previous studies had reported that the integrin signaling pathway was involved in a diverse set of cellular functions including the initiation, progression and metastasis of tumors^26^. In central nervous system, phosphorylation of eIF2 terminates global protein translation and induces apoptosis, and dysfunction of eIF2 signaling enables cells to escape from programmed cell death and thus induce cancer^27^. Previous studies also suggested that failure to keep PKA under control may lead to neoplastic transformation and tumor growth^28^. Neurotrophic Factor and growth factor activations are also involved in eIF4-p70s6k and ERK5 signaling pathways, which could enhance cell proliferation in brain tumors. PI3K/Akt pathway is a key determinant of aggressive tumors, and a major target for novel anti-cancer therapies^29^. The diverse functional modules enriched in the REST associated transcripts implied that REST may coordinate with these modules to initiate and promote glioblastoma.

### MicroRNA expression pattern and REST

Because several miRNAs are known to be involved in GBM, correlation between miRNAs and REST score was investigated in the next step. Figure 3C showed that numerous miRNAs were significantly correlated with REST activity, either positively or negatively. Negative correlation was observed between the REST score and several miRNAs, with the miR-7^30^ and miR-219^31^ having the tumor suppressor role where miR-7 was known to inhibit tumor angiogenesis in GBM^32^. We also observed decreased miR-124a expression in REST enhanced GBM patients, which was consistent with a previous finding that miR-124a was down-regulated by REST in glioblastoma^33^. On the other hand, several miRNAs were up-regulated in REST enhanced GBM including miR-221, miR-222, miR-34a, and miR-204. This was in line with previous findings where high expression of miR-221 and miR-222^34^,^35^ were reported to promote GBM invasion and angiogenesis by targeting TIMP2 and PTPμ.

To explore the functional interplay between miRNA and mRNA during REST regulation, we downloaded experimentally validated mRNA targets using the miRWalk database^36^ for the identified miRNAs in Figure 3C. Among the 77 genes in the EXPREST signature, 30 genes were validated targets to the identified miRNAs, which is much higher than expected (Fisher exact test p = 1.58 × 10^−4^). Looking at the association between mRNAs and REST score, correlation p values from the validated miRNA targets were significantly smaller than those from non-target mRNAs (Kolmogorov–Smirnov test p = 2.20 × 10^−16^). The enrichment of validated miRNA targets in the EXPREST signature as well as in the REST score-associated mRNA molecules suggested miRNA play important roles in regulating REST activity.

### REST-associated mutations

We further investigated the impact of REST activity on mutational landscape. We observed that several mutations were associated with decreased REST activity such as IDH1, ATRX, SPTA1, and TP53 (Fig. 3D). IDH1 mutations had been reported as a prognostic factor for better survival^37^ and a defining characteristic of the proneural subtype^21^ in GBM. ATRX mutations were found to be restricted to IDH1/2 mutated glioma tumors and were also associated with better prognosis^38^. Interestingly, GBM tumors of proneural subtype were associated with lowered REST activity (Fig. 2C) in our analysis, suggesting the interplay among IDH1 and ATRX mutations with REST activity during neuronal development. Both TP53 and SPTA1 were frequently mutated in GBM but there was no previous studies reporting their functional association with REST. There were also several mutations associated with increased REST activity including NF1 and LAMA1. NF1 was reported as being significantly mutated in GBM, but its occurrence was mutually exclusive to IDH1 mutation^39^, which in part explained their opposite association with REST. Mutations in LAMA1 was previously found to cause cerebellar dysplasia and cysts^40^. Further, LAMA1 overexpression was observed in GBM relative to non-neoplastic brain tissue^41^, suggesting a synergistic effect between REST and LAMA1 during tumorigenesis.

### REST enhanced cell lines are more sensitive to IGF1R inhibition

**Figure 4:**
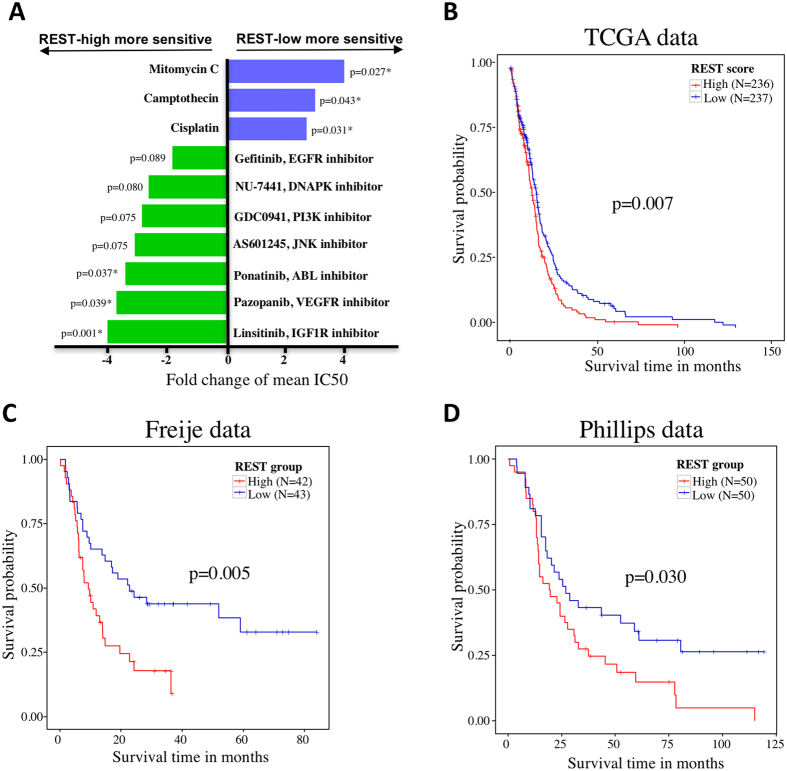
Therapeutic and prognostic relevance of the EXPREST signature. (**A**) Cell lines in the REST-high group (defined based on the median REST score as the cutoff) were more sensitive to IGF1R, VEGFR and ABL inhibitors while more resistant to cytotoxic drugs including Mitomycin, Camptothecin and Cisplatin. X-axis showed the fold change of mean IC_50_ values. (**B**) Kaplan-Meier curves for TCGA GBM tumors demonstrating prognostic value of the EXPREST signature. (**C**) Kaplan-Meier curves for the Freije data as an independent validation. (**D**) Kaplan-Meier curves for the Phillips data as an independent validation.

We posited that differences in REST activity would confer distinct patterns of therapeutic response. To test this hypothesis, we assessed the association between the EXPREST signature score and drug response (IC_50_ values) using public drug sensitivity database (the Genomics of Drug Sensitivity in Cancer^42^). Linsitinib, an IGF1R inhibitor currently in Phase III clinical trials for adrenocortical carcinoma, emerged as the top hit associated with enhanced activity in REST-high cell lines (Fig. 4A). IGF1R inhibition was previously reported to be effective in a subset of pediatric glioblastoma cell lines^43^. Our result further demonstrated REST enhanced GBM tumors may benefit more from IGF1R inhibition. In addition to IGF1R inhibition, cell lines with high REST activity were also more sensitive to VEGFR inhibition targeted by Pazopanib. Ponatinib, a tyrosine-kinase inhibitor for ABL approved to treat chronic myeloid leukemia also showed increased sensitivity in REST enhanced samples (Fig. 4A). In contrast, cell lines with low REST activity were more sensitive to cytotoxic drugs including Mitomycin, Camptothecin and Cisplatin.

### High REST activity is associated with worse clinical outcome

Increased REST activity promotes tumorigenicity by inhibiting neural differentiation and thus may negatively impact patient survival and prognosis^12^. We thus investigated whether our REST signature was able to predict clinical outcome. We grouped the patients into low and high REST activity group based on the REST score using median as the cutoff. The group with high REST activity was found to have significantly shorter overall survival compared to the low REST activity group in TCGA GBM patients (log rank test p = 0.007, hazard ratio HR = 1.35, 95% confidence interval C.I. = 1.09~1.67, Fig. 4B). For the robustness of data analysis, we also performed a further study treating the REST score as a continuous variable. Cox regression analysis demonstrated that REST score was significantly associated with poor survival (p = 0.013) with a unit increase in REST score leading to a hazard ratio of 1.14 (CI = 1.03~1.27).

We then validated our REST signature on two independent gene expression data sets, namely, the Freije data set (accession number GSE4412) and the Phillips data set (accession number GSE4271). Each of the validation samples was assigned a prognostic score measuring REST activity based on our REST signature (see Methods). For the Freije data set, high REST activity was found to be associated with worse overall survival (log rank test p = 0.005, HR = 2.17, CI = 1.26~3.71, Fig. 4C). Similar result was found for the Phillips data set (log rank test p = 0.030, HR = 1.76, CI = 1.05~2.93, Fig. 4D).

## Materials and Methods

### Data Collection

Microarray gene expression (HT HG U133A) data for the GBM cell line data was downloaded from the Genomics of Drug Sensitivity in Cancer website (http://www.cancerrxgene.org/). Cell line information including tissue type and cell line name was downloaded from the COSMIC database (ftp://ftp.sanger.ac.uk/pub/CGP/cell_lines_project/data_export/). There were 47 central nervous system (CNS) samples derived from GBM patients. IC50 values were extracted for these cell lines^44^. IC50 was defined as the drug concentration needed to kill 50% of cell lines through a cell line viability assay. The GBM patient data was downloaded from TCGA data portal (https://tcga-data.nci.nih.gov/tcga/). There were 473 tumor samples with both clinical information and RNA-Seq expression data (quantified and normalized by the RSEM software^45^). Among the 473 TCGA tumor samples, 236 samples had mutation data and 433 samples had microRNA expression data. Two additional microarray data sets were downloaded from Gene Expression Omnibus (GEO) for independent validation. The Freije data set (accession number GSE4412) contained 85 glioma samples. The Phillips data set (accession number GSE4271) contained 100 glioma samples. Both data sets were profiled with the Affymetrix HG-U133A/B platforms and normalized by the MAS5 algorithm^46^. Gene expression data for healthy tissue dissected from different regions were downloaded from GEO with accession GSE43346 (Affymetrix U133 plus 2.0, also normalized by MAS5 algorithm). Overall survival with censoring status was extracted from the GEO website for both validation sets.

### REST Signature Development

Since public signature developed from cell line data showed inconsistent correlation with REST expression in cell line and GBM patient data (Supplementary Table S1), we resorted to develop a GBM-specific REST signature. Using a seed guided search similar to the approach in Mak *et al.*^25^, we first identified genes associated with REST expression by computing the Pearson’s correlation in both CNS cell line and GBM patient expression profiles. To select a subset of genes showing strong correlation to REST expression as well as to shrink the gene list to a manageable size, we filtered out genes with opposite correlation sign between cell line and GBM patient data. With an absolute correlation cutoff of 0.3, 77 genes were identified as the GBM REST signature consisting of 9 genes with negative correlation (RESTless-type) and 68 genes with positive correlation (REST-type). We referred this expression-based REST signature as EXPREST. To quantify the REST potential of each sample using the derived signature, a REST score (*S*_*i*_) for sample *i* can be calculated as following:


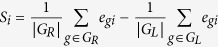


Here G_R_ represents the set of REST-type genes (with positive correlation to REST) and G_L_ represents the set of RESTless-type genes (with negative correlation to REST). |G_R_| and |G_L_| denotes the number of REST-type and RESTless-type genes in the signature. e_gi_ is the expression value for gene g in sample i. Intuitively, the REST score is the mean expression of REST-type genes subtracted by the mean expression of the RESTless-type genes. Hence, a large REST score means REST activity is enhanced. REST high and low groups used in Kaplan-Meier survival analysis was defined based on the median cutoff of REST score.

To illuminate the functional involvement of our EXPREST signature, pathway enrichment analysis was performed using Ingenuity Pathway Analysis (IPA^®^, QIAGEN Redwood City, www.qiagen.com/ingenuity) and visualized with the Cytoscape software^47^.

### Statistical Analysis

To identify genomic features associated with REST activity, different statistical approaches were used depending on the nature of genomic data. In particular, nonparametric Spearman rank correlation (ρ) was calculated between REST score and gene expression (microarray) or protein expression (RPPA) data. Student’s t test was used to identify mutations associated with REST activity. Student’s t test was also used to investigate if REST groups (low versus high) were associated with IC50 values. Here the response variable was assumed to be independently drawn from normal distribution with equal variance between groups. ANOVA was used to assess the association between REST and GBM molecular subtypes and Tukey’s honest significant difference (HSD) test was used for subsequent post-hoc analysis. Log-rank test was used to test if REST score was associated with overall survival. Cox regression was used to estimate hazard ratio (HR) for both binary and continuous covariates. This assumed the survival data had proportional hazards and random censoring. To correct for multiple testing, the Beta-Uniform Mixture (BUM) model was used to estimate False Discovery Rate (FDR)^48^ and the Benjamini-Hochberg procedure^49^ was used to estimate adjusted p value. All statistical analysis was performed using the R software^50^.

## Discussion

Glioblastoma is non-capsulated brain tumor typically refractory to conventional therapies. Development of novel therapeutics, especially targeted therapy addressing patients’ unique tumors is critical in overcoming this malicious disease. Previous studies had attributed therapeutic failure to cancer stem cells which is able to initiate tumor recurrence, even when the tumor mass is surgically removed^51^. REST has been found to be a potent regulator maintaining the self-renewal process of cancer stem cells in GBM and thus a good candidate for therapeutic interventions^52^. Here we employed an integrated genomic approach to characterize REST activity and investigate its relevance to therapeutic targeting in brain tumors. Our EXPREST signature was specifically adapted to the unique nature of GBM patients and was able to accurately quantify the REST activity in both cell line models and patient tumors.

Using the EXPREST signature as the apparatus for REST quantification, we observed that GBM tissue had elevated REST activity than adjacent normal tissue. Therefore, REST demonstrated oncogenic properties in GBM, which was consistent with previous findings^53^. In further characterizing the molecular properties associated with REST activity, we found that patients with enhanced REST activity were enriched with the mesenchymal subtype of GBM. Previous study of the mesenchymal subtype reported high expression of mesenchymal markers including CHI3L1 and MET^21^, which was reminiscent of the EMT process linked to dedifferentiated and transdifferentiated tumors^23^. We further validated this discovery by observing a strong positive correlation between REST activity and EMT potential. Interestingly, baseline REST activity for differentiated brain tissues appeared to be suppressed compared to other non-neural tissues among healthy population. For non-neural tissues, inactivation of REST activity may lead to cancer including breast^14^ and small cell lung cancer^54^. The seemingly paradoxical role of REST as both a tumor suppressor in non-neural tissue and oncogene in neural tissue demonstrates the complex nature of REST regulation^55^.

Our genomic characterization of REST for the first time revealed global transcriptomic deregulations in glioblastoma. This discovery suggests REST might play wider roles than we already know in regulating neural differentiation. In particular, the enriched pathways including PKA signaling, EIF2 signaling, PI3K/AKT signaling, G2/M DNA damage checkpoint and Wnt/Ca+ signaling pointed the widespread signaling networks related to REST through either direct or indirect regulations. Interestingly, we also identified several REST related miRNAs including miR-124a already reported in the literature and a few novel miRNAs warranting further investigation.

Our study also demonstrated direct therapeutic relevance of REST targeting in glioblastoma. Prognostically, enhanced REST activity was correlated with reduced overall survival in both our primary dataset and two independent validation sets. Therapeutically, we identified several drugs being more sensitive to REST enhanced preclinical cell line models including Linsitinib, Pazopanib, and Ponatinib. For cell lines with low REST activity, cytotoxic drugs including Mitomycin, Camptothecin and Cisplatin appeared to be more effective. The differential sensitivity based on REST activity suggests REST is a promising biomarker to stratify GBM patients into different risk groups for individualized cancer treatments.

## Additional Information

**How to cite this article**: Liang, J. *et al.* An expression based REST signature predicts patient survival and therapeutic response for glioblastoma multiforme. *Sci. Rep.* **6**, 34556; doi: 10.1038/srep34556 (2016).

## Supplementary Material

Supplementary Table S1

Supplementary Table S2
