# Supplementary material for: An expression based REST signature predicts patient survival and therapeutic response for glioblastoma multiforme
Source: Sci Rep. 2016 Oct 4;6:34556. doi: 10.1038/srep34556 (PMC5048293; doi:10.1038/srep34556)
Supplement: Supplementary Table S1 [file srep34556-s1.doc]

**An expression based REST signature predicts patient survival and therapeutic response for glioblastoma multiforme**

Jianfeng Liang ^1^, Qinghua Meng ^2^, Wanni Zhao ^3^, Pan Tong ^4^, Ping Li ^5^, Yuanli Zhao ^1, 6^, Xiaodong Zhao ^7^*, Hua Li ^7^*

^1^ Department of Neurosurgery, Peking University International Hospital, Beijing 102206 China;

^2^ Department of Nutrition, Jinan Central Hospital Affiliated to Shandong University, Jinan, Shandong Province 250013, China;

^3^ Peking University China-Japan Friendship School of Clinical Medicine，Beijing 100029, China;

^4^ Department of Bioinformatics and Computational Biology, The University of Texas M. D. Anderson Cancer Center, Houston, TX 77030, USA;

^5^ Department of Hematology, Tongji Hospital of Tongji University, Shanghai, 200065, China;

^6^ Department of Neurosurgery, Beijing Tiantan Hospital, Capital Medical University, Beijing 100050, China;

^7^ Bio-ID Center, School of Biomedical Engineering, Shanghai Jiao Tong University, Shanghai, 200240, China.

* To whom correspondence should be addressed. Xiaodong Zhao, Email: xiaodongzhao@sjtu.edu.cn; Hua Li, Email: kaikaixinxin@sjtu.edu.cn.

|  | **GBM Cell Line** | | **GBM Patients (TCGA)** | |
| --- | --- | --- | --- | --- |
|  | **Correlation** | **p value** | **Correlation** | **p value** |
| HBA1 | -0.13332 | 0.37164 | -0.07929 | 0.51721 |
| HBA2 | -0.00678 | 0.96393 | 0.02844 | 0.81655 |
| SNAP25 | 0.00513 | 0.97270 | -0.30425 | 0.01103 |
| KCNB1 | 0.01319 | 0.92986 | 0.12991 | 0.28736 |
| AP3B2 | 0.02305 | 0.87780 | -0.08212 | 0.50232 |
| STMN3 | 0.06623 | 0.65824 | -0.20033 | 0.09886 |
| VGF | 0.06721 | 0.65352 | 0.03753 | 0.75949 |
| CPLX2 | 0.08600 | 0.56546 | -0.19193 | 0.11413 |
| PGBD5 | 0.12885 | 0.38804 | -0.10939 | 0.37093 |
| SCGB1D2 | 0.14888 | 0.31792 | -0.19169 | 0.11459 |
| BSN | 0.19711 | 0.18417 | 0.03304 | 0.78756 |
| CHGB | 0.24090 | 0.10285 | -0.23208 | 0.05500 |
| SYP | 0.24570 | 0.09597 | -0.22065 | 0.06846 |
| RTN2 | 0.39919 | 0.00544 | -0.33389 | 0.00505 |
| SCAMP5 | 0.40815 | 0.00440 | -0.20075 | 0.09814 |
| MAPK8IP2 | 0.48593 | 0.00053 | -0.23642 | 0.05049 |
| MMP24 | 0.48639 | 0.00053 | -0.31446 | 0.00850 |
| CPLX1 | NA* | NA | -0.22771 | 0.05987 |
| DISP2 | NA | NA | -0.22558 | 0.06237 |
| GOLGA7B | NA | NA | -0.21779 | 0.07223 |
| RLTPR | NA | NA | -0.25631 | 0.03352 |
| RUNDC3A | NA | NA | -0.27010 | 0.02480 |
| TMEM145 | NA | NA | -0.13690 | 0.26199 |
| TMEM198 | NA | NA | -0.27551 | 0.02195 |

Table S1: Correlation between REST and target genes from public REST signature. Among the 24 public REST signature genes, only two genes were negative correlated with REST expression in GBM cell line data.

NA*: expression data not available in cell line microarray data
